# NGT-3D: a simple nematode cultivation system to study *Caenorhabditis* *elegans* biology in 3D

**DOI:** 10.1242/bio.015743

**Published:** 2016-03-23

**Authors:** Tong Young Lee, Kyoung-hye Yoon, Jin Il Lee

**Affiliations:** Division of Biological Science and Technology, College of Science and Technology, Yonsei University, Wonju 220-710, South Korea

**Keywords:** *C. elegans*, Three dimensions, Reproduction

## Abstract

The nematode *Caenorhabditis* *elegans* is one of the premier experimental model organisms today. In the laboratory, they display characteristic development, fertility, and behaviors in a two dimensional habitat. In nature, however, *C. elegans* is found in three dimensional environments such as rotting fruit. To investigate the biology of *C. elegans* in a 3D controlled environment we designed a nematode cultivation habitat which we term the nematode growth tube or NGT-3D. NGT-3D allows for the growth of both nematodes and the bacteria they consume. Worms show comparable rates of growth, reproduction and lifespan when bacterial colonies in the 3D matrix are abundant. However, when bacteria are sparse, growth and brood size fail to reach levels observed in standard 2D plates. Using NGT-3D we observe drastic deficits in fertility in a sensory mutant in 3D compared to 2D, and this defect was likely due to an inability to locate bacteria. Overall, NGT-3D will sharpen our understanding of nematode biology and allow scientists to investigate questions of nematode ecology and evolutionary fitness in the laboratory.

## Abstract (precis)

**Summary:** We have designed a three dimensional nematode habitat for use in the lab, called NGT-3D, where growth, fertility and lifespan are comparable with the traditional two dimensional lab environment.

## INTRODUCTION

The nematode *Caenorhabditis elegans* has been one of the most useful genetic model organisms in biology leading to several seminal discoveries. The wild-type *C. elegans* Bristol strain N2, originally obtained from a mushroom compost pile in England, has been cultivated in the laboratory for decades (Ferris and Hieb, 2015), and maintained on the surface of a specialized agar called nematode growth media (NGM) that supports the growth of both nematode and its food, *E. coli* bacteria. Experiments on NGM have led to the discovery of a worm that displays complex and curious sensory behaviors along with memory that draws parallels with higher metazoans.

Despite the plethora of studies in the laboratory, little is known about worm biology and ecology in their native environments. Only recently have we learned that *C. elegans* and other Rhabditid nematodes can be found thriving in rotting fruit or vegetative matter (Barrière and Félix, 2005). Thus, in nature worms grow in three dimensions rather than the two-dimensional plates in the laboratory. This brings to question the relevance of the developmental, physiological, and behavioral phenotypes associated with *C. elegans* grown on 2D NGM. For instance, mutants with serious physiological deficits, such as severe loss of muscle function (Epstein et al., 1974) and synaptic activity (Richmond et al., 1999) that should be detrimental to survival in the wild, live and breed quite normally in the 2D laboratory environment.

We sought to design a laboratory simulation of natural conditions to study how worms interact with the surrounding environment at the genetic level. Although other systems were designed to observe acute *C. elegans* behavior in 3D (Beron et al., 2015; Kwon et al., 2013), our goal was to cultivate worms in 3D over their life cycle. Our 3D natural simulated habitat for *C. elegans* is simple and easy for other scientists to replicate in the laboratory, allows for near-normal growth of *C. elegans* over several generations, and can be used to show the fitness contribution to reproduction that certain genes confer to worms.

## RESULTS AND DISCUSSION

The standard NGM plate presents several ideal features including easy mass production, ease of use on a stereomicroscope, plentiful bacterial growth, and, of course, support for worm growth. Our first goal was to incorporate some of these important features into a 3D nematode cultivation system, namely: (1) Worms can move freely in 3D, (2) support OP50 strain *E. coli* growth, (3) worm 3D behavior can be visualized, and (4) easy to manufacture and maintain. To aid in the visualization of worms in 3D, we decided to use Difco granulated agar due to its translucent appearance compared to the regular Bactoagar. We found that the ideal concentration for worm movement was at agar concentrations between 0.4 to 0.6%. Above 0.6%, worms moved slowly in 3D, and below 0.4%, the agar constitution became somewhat watery, compromising normal movement. Therefore, we settled on a concentration of 0.5% granulated agar.

**Fig. 1.**
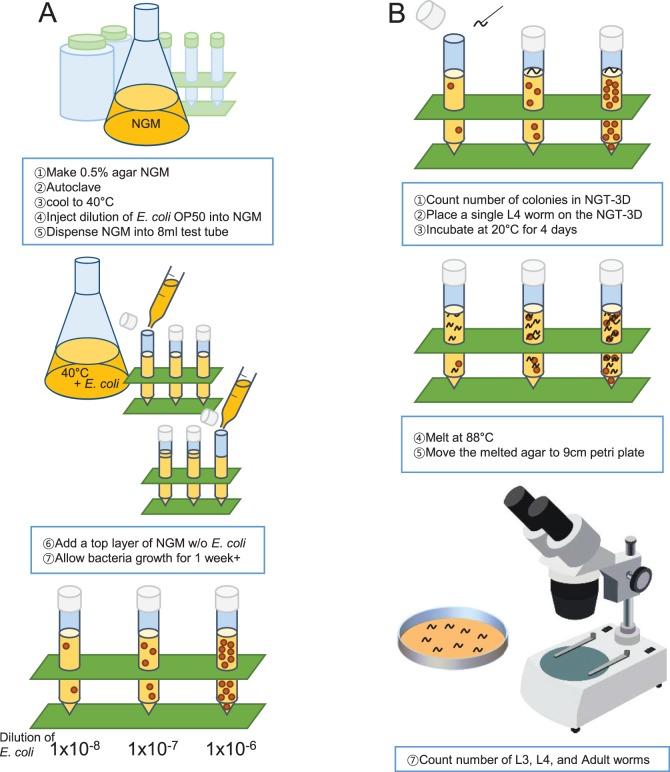
**Design and strategy for the NGT-3D experiment.** (A) Illustration of NGT-3D production. (B) Experimental strategy and method to assess reproductive fitness by relative brood size.

Our method to build a 3D worm cultivation chamber is outlined in Fig. 1A. To house the agar in 3D, we used a standard 8 ml test tube due to its clarity. OP50 bacteria growth immersed in the NGM requires seeding before the agar hardens; however the high temperature of the liquid agar can also kill the bacteria. After pouring the hot agar into the test tube, we allowed the temperature to drop to just below 40°C, then injected OP50 liquid culture into liquid agar, mixed, and allowed the agar to harden. After several days, small spheroid colonies with diameters between 1.5 to 3.5 mm appeared throughout the agar. Adjusting the dilution of the OP50 liquid culture regulates the number of colonies that grow in the NGM (Fig. 1A, bottom). We found dilutions of 1×10^−7^ produced about 12 colony forming units (CFUs) in each tube and 1×10^−6^ produced about 120 colony forming units. We decided to term this ‘nematode growth tube-3D’ or NGT-3D for short. Finally, we placed a single worm on the top of the agar. The worm can easily make its way into the agar moving from colony to colony, eating, growing and reproducing. One caveat was that if an OP50 colony was near or at the surface of the NGT-3D, the worm might reside only at the 2D surface of the agar. To prevent growth of OP50 colonies near the surface, we poured agar over the top of the initial agar-OP50 mix immediately after it began to harden, forming a thin bacteria-free layer at the top of the NGT-3D.

We first wanted to confirm whether *C. elegans* growth and reproduction was comparable in NGM plates and the NGT-3D. To test this, single wild-type N2 L4 stage larvae were placed in NGM plates or in NGT-3D, left for four days and allowed to lay eggs (Fig. 1B). To count progeny worms, the agar was melted at 88°C and poured into large petri plates, which kills the worms but keeps their bodies intact. Since it is not possible to remove the original P_0_ generation worm, we let F_1_ worms grow for four days and counted only adult, L4, and L3 worms of the F_1_ generation and the P_0_ adult worm. Younger worms were ignored as to not confuse F_1_ and F_2_ generations. Therefore, we are measuring the ‘relative’ brood size produced that have reached at least the L3 larval stage after four days, rather than the total brood size over the life of the worm.

**Fig. 2.**
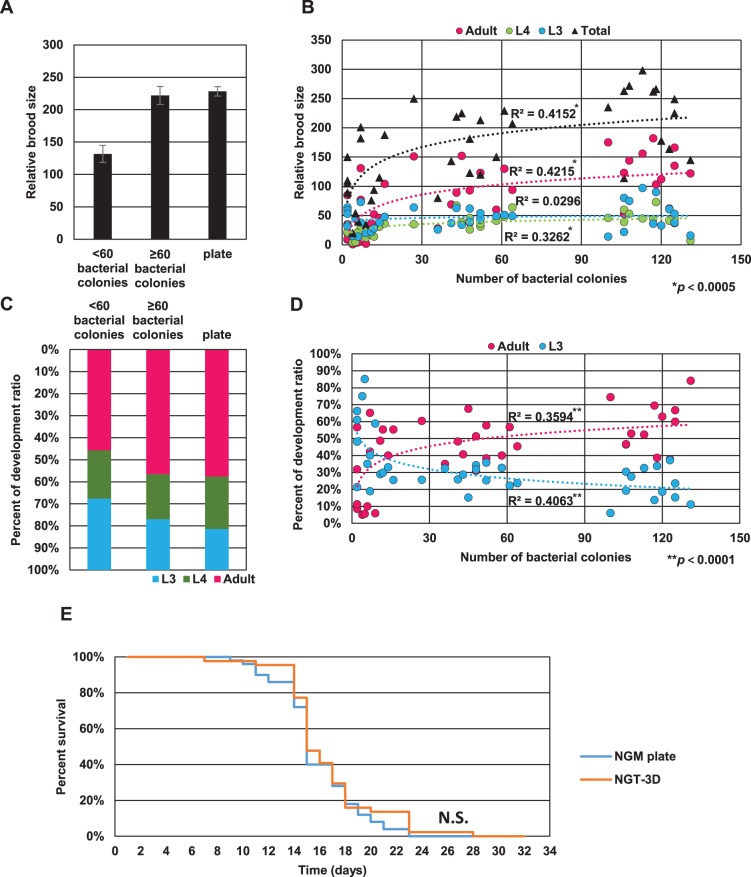
**Fertility, growth and lifespan of wild-type *C. elegans* on NGM plates and NGT-3D.** (A) Relative brood size of wild-type worms in NGT-3D with fewer than 60 OP50 colonies, greater or equal to 60 colonies, or on 2D NGM plates. Error bars indicate standard error. (B) Scatter plot of worm populations in the L3, L4 and adult developmental stages and total worms against the number of colonies in NGT-3D. Logarithmic regressions are plotted as dotted lines and the correlation coefficient R^2^ is calculated. Significance of regression is indicated. (C) Percent of F_1_ generation worms in the L3, L4 or adult developmental stage in NGT-3D with fewer than 60 OP50 colonies, greater than or equal to 60 colonies, or on 2D NGM plates. (D) Scatter plot of the percent of worms in the L3 and adult developmental stages and total worms against the number of colonies in NGT-3D. Logarithmic regressions are plotted as dotted lines and the correlation coefficient R^2^ is calculated. Significance of regression is indicated. (E) Survival curve of wild-type *C. elegans* in NGT-3D or NGM plates. N.S. indicates not significantly different calculated by log-rank test.

We found that relative brood size varied in NGT-3D culture conditions, depending on the number of OP50 colonies that formed within each NGT-3D. When the number of OP50 colonies in the NGT-3D happened to be 60 or greater, the relative brood size was comparable (222±14) to 2D growth on NGM plates (228±7; Fig. 2A). These progeny worms also had comparable growth rates, with the majority of worms reaching adulthood on both NGM plates and NGT-3D (Fig. 2C). However, when under 60 colonies were present, the brood size in NGT-3D dropped dramatically (132±13) with only 46% of the worms reaching adulthood. This is not due to the depletion of bacteria, as none of the bacterial colonies are completely consumed. Thus, conditions for worm cultivation in NGT-3D are similar to cultivation on NGM plates when more than 60 bacterial colonies are present, and a dilution of 1×10^−6^ OP50 *E. coli* is sufficient to produce this condition.

We more carefully examined the reproduction and growth rates in NGT-3D by plotting the number of L3, L4, adult or total worms produced in the F_1_ generation against number of OP50 colonies (Fig. 2B). The scatter plot allowed us to observe trends in the data, which we further analyzed by a logarithmic regression analysis. We found that relative brood size increases with the number of OP50 colonies until it reaches saturation (R^2^=0.42), indicating that the availability of bacteria in NGT-3D is an important factor in worms to grow in NGT-3D. Interestingly, we also observed that the number of adult and L4 worms increased with the number of bacteria colonies (R^2^=0.42 and R^2^=0.33, respectively), whereas the number of L3 worms did not (R^2^=0.030).

To explain this observation, we plotted the percentage of L3 or adult worms against number of OP50 colonies (Fig. 2D) and again analyzed the scatter plot by logarithmic regression analysis. We found a relatively strong correlation between number of colonies and percentage of adults (R^2^=0.36) and an inverse correlation (R^2^=0.41) between percent L3 and number of colonies, indicating that fewer bacterial colonies results in a younger F1 worm population. This could indicate either slower growth rates or a delaying of reproduction when bacterial colonies are scarce.

A previous study showed that when worms were cultivated in soil or sand, lifespan was significantly shortened (Van Voorhies et al., 2005). We wondered whether growth in NGT-3D compromised wild-type *C. elegans* lifespan. Worms grown on NGT-3D lived an average of 15.6±3.6 days compared to 14.8±3.1 on 2D NGM. Analysis of survival curves showed no significant difference between lifespan on NGT-3D and NGM plate (Fig. 2E). Thus, lifespan of *C. elegans* is also comparable between NGT-3D and 2D NGM plates.

**Fig. 3.**
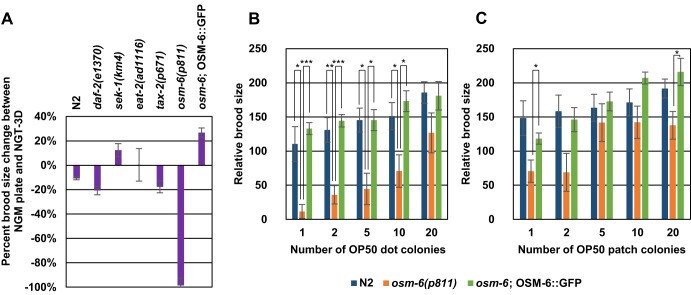
**Animals defective in *osm-6* show compromised brood size in NGT-3D.** (A) Percent difference between relative brood size in NGT-3D with more than 40 OP50 colonies and NGM plates is shown for N2 wild-type and several types of mutants. Sample size: N2, *n*=22; *daf-2, n*=8; *sek-1, n*=6, *eat-2, n*=5; *tax-2, n*=6; *osm-6, n*=5; *osm-6;* OSM-6::GFP, *n*=6. (B,C) Relative brood size was determined for N2 and *osm-6* mutants and *osm-6*;OSM-6::GFP rescue strain on OP50 dot plates (B) and OP50 patch plates (C) with varying numbers of bacterial colonies. See Materials and methods for explanation of the types of plates. Sample size *n*=5 for all. Error bars indicate standard error. Significance is calculated by Student's *t*-test. *P<0.05; **P<0.01; ***P<0.001.

We were curious to understand any genetic factors that may cause differences in growth or reproduction in 3D compared to 2D. We investigated this by observing the growth and reproduction of several candidate mutants in the NGT-3D containing plenty of OP50 colonies. We first tested mutants that affect two conserved signaling pathways, insulin signaling and MAP kinase signaling. The *daf-2* gene encodes the receptor for an insulin-like growth factor (Kimura et al., 1997). Mutants of *daf-2* are significantly long-lived compared to wild-type worms (Kenyon et al., 1993). However, one study showed that *daf-2* mutants were short-lived compared to wild-type worms when cultivated in natural soil (Van Voorhies et al., 2005). Mutants of *daf-2* grown in NGT-3D showed little change in relative brood size compared with brood sizes on NGM plates (Fig. 3A), comparable to what is observed in N2 wild type. This indicates that insulin signaling pathways may be dispensable for cultivation in 3D. The *sek-1* gene encodes the MAPK kinase protein important for MAP kinase signaling in worms (Tanaka-Hino et al., 2002). *sek-1* mutants have slightly compromised growth and development, and are easily susceptible to infection (Kim et al., 2002). However, the relative brood size of *sek-1* mutants in NGT-3D also shows little change compared to cultivation on NGM plates (Fig. 3A). Hence, neither insulin nor MAP kinase signaling seem to be important for differences we observe in 3D vs 2D growth and fertility.

We also examined *eat-2* mutants that exhibit decreased pharyngeal pumping. This defect results in decreased food intake for worms which slows growth and decreases fertility (Raizen et al., 1995). Cultivation in 3D conditions, however, did not result in any significant changes in relative brood size (Fig. 3A). Thus, slower feeding rate does not seem to disrupt growth in 3D conditions.

Although bacterial availability is not an issue in NGT-3D, the distance between colonies may pose a challenge for *C. elegans* to find a meal. In particular, sparse colonies in NGT-3D may make it more difficult for worms to find food. This is normally not a problem for nematodes on a 2D plate with only two dimensions to search, but can pose a major challenge in 3D with an extra dimension that needs to be surveyed. *C. elegans* uses ciliated amphid sensory neurons in its head to sense odors, chemicals, temperature and a variety of other environmental cues to find proper habitats and sources of food (Bargmann, 2006). Moreover, *osm-6* mutants, which are defective for the development of ciliated sensory neurons, were defective at food search behavior (Gray et al., 2005). We tested fertility in NGT-3D in the *osm-6* mutant, and also a mutant of *tax-2* which encodes a subunit of a cyclic nucleotide-gated receptor that is required for the function of several of the amphid neurons (Coburn and Bargmann, 1996; Coburn et al., 1998).

*osm-6* mutants show normal brood sizes similar to N2 animals on a 2D NGM plate (data not shown). However when cultivated in NGT-3D, *osm-6* mutants produce almost no young even when more than 40 OP50 colonies are present (Fig. 3A). This experiment proves the importance of sensory neurons to the reproductive fitness of the worm, which cannot be demonstrated on a normal NGM plate. Cultivation of *tax-2* mutants in 3D showed similar changes in fertility compared to wild-type worms.

The *osm-6* gene encodes a protein which is a component of the intraflagellar transport particle required for proper sensory cilium structure (Collet et al., 1998; Ou et al., 2007). Introduction of a functional OSM-6::GFP transgene into the genome of *osm-6* mutants rescues a sensory neuron dye-labeling defect that is a result of abnormal sensory cilium (Collet et al., 1998). Using this *osm-6;* OSM-6::GFP strain, we determined relative brood size on NGM plates and NGT-3D. We found that OSM-6::GFP expression in *osm-6* mutants can restore the low brood size defect in NGT-3D, albeit slightly increase fertility on NGT-3D (Fig. 3A). Thus, low brood size in *osm-6* mutants is a result of loss of OSM-6 function and associated sensory cilia defects.

To determine whether *osm-6* fertility is compromised due to an inability to navigate towards bacteria, we challenged *osm-6* mutants on 2D NGM plates that had either 3-5 mm diameter ‘patches’ of OP50, or smaller 1-2 mm diameter ‘dots’ of OP50. Each plate had 1, 2, 5, 10, or 20 OP50 dots or patches spread evenly across the plate, and we assessed the relative brood size of N2, *osm-6* mutants, and the *osm-6*;OSM-6:GFP rescue strain. We found that wild-type worms reproduced fine in nearly all conditions (Fig. 3B,C). However, the reproductive fitness of *osm-6* mutants was lower in OP50 patches, and significantly lower in 1 to 10 OP50 dots (Fig. 3B,C). We also performed this experiment in the *osm-6* rescue strain, and found that expression of a wild-type copy of the *osm-6* gene can restore low brood sizes on both patch and dot plates in *osm-6* mutants (Fig. 3B,C). From this data, we speculate that *osm-6* is necessary for worms to navigate complex environments to find food, bear young, and reproductively compete with other worms and organisms.

Overall, we have successfully developed and tested a standard method to cultivate worms in 3D. Unlike cultivation in soil or sand in which worm lifespan significantly decreases (Van Voorhies et al., 2005), wild-type *C. elegans* shows no difference in lifespan on NGT-3D compared to NGM plates (Fig. 2E). Cultivation in soil or sand is a closer simulation to the natural nematode habitat than our agar matrix. Still, the NGT-3D is an better reflection of natural conditions for *C. elegans* compared to the standard 2D NGM plate, and we show that here worms can develop, reproduce, and age similarly in either environment. It also poses challenges such as finding bacteria in three dimensions that are quite relevant to the worm in natural environments. Hence, the NGT-3D may be useful to determine the reproductive fitness levels of different genetic backgrounds. In addition, we are currently examining how *C. elegans* behavior differs in 3D vs 2D over several generations, and we hope the development of the NGT-3D will encourage others to investigate the behavior, physiology, development and lifespan of worms in a more naturally simulated environment within the laboratory.

## MATERIALS AND METHODS

### Maintenance of *C. elegans*

All *C. elegans* strains were maintained at 20°C on NGM seeded with OP50 strain *E. coli* (Lewis and Fleming, 1995). Bristol N2 wild type, PR811 *osm-6(p811)*, PR671 *tax-2(p671)*, CB1370 *daf-2(e1370)*, KU4 *sek-1(km4)*, and DA1113 *eat-2(ad1116)*, and SP2101 *ncl-1(e1865) unc-36(e251); osm-6 (p811); mnIs17*[(*p*)*osm-6::*OSM-6::GFP] strains were provided by the Caenorhabditis Genetic Center (Minnesota, USA).

### Nematode growth media plates

Standard techniques were used to make NGM agar plates (Lewis and Fleming, 1995) except that Difco Granulated Agar was used as the solidifying agent (20 g in 1 litre media). For OP50 dot plates, a sterile toothpick dipped in an OP50 culture was used to place from 1 to 20 evenly spaced dots of OP50 across a 5.5 cm fresh NGM plate and incubated overnight at 37°C, resulting in colonies with diameters between 1-2 mm of increased density with more dots. For OP50 patch plates, 0.5 µl of OP50 was used to place from 1 to 20 evenly spaced patches across a 5.5 cm fresh NGM plate, resulting in colonies with diameters between 3-5 mm of increased density with more patches.

### Nematode growth tube-3D

The media for NGT-3D is similar to NGM. For 1 litre of media, 3 g NaCl, 5 g Difco Granulated Agar, 2.5 g peptone, and 975 ml water was autoclaved at 121°C for 15 min. After cooling down to 55°C, 1 ml of 1 M CaCl_2_, 1 ml of 1 M MgSO_4_, 1 ml of 5 mg/ml cholesterol in ethanol, and 25 ml of 1 M KPO_4_ buffer was added. To seed NGT-3D, an overnight culture of OP50 was serially diluted with a 0.85% NaCl solution. After the temperature of the media cooled to 40°C, the dilution was added directly into the media. For our brood size and development assays we used diluted 30 ml of 1×10^−6^, 1×10^−7^ or 1×10^−8^ and 15 ml 1×10^−6^ OP50 dilutions. After mixing, the media was poured into 8 ml clear plastic test tubes (Stockwell Scientific) and was briefly left to harden. A layer of NGM agar without OP50 was poured on top to prevent colonies forming too close to the surface and left for at least 10 days for bacterial growth.

### Relative brood size and development

L4 worms were picked, and bacteria was washed off by placing the worm on a non-seeded plate for several minutes and repeated twice. The cleaned worm was placed on either an NGM plate or NGT-3D with a platinum wire pic, and incubated for 96 h at 20°C. To count ‘relative’ brood size, NGT-3D and the NGM plate were melted in an 88°C water bath and poured into 9 cm plates. Worms die but remain intact. When counting, only adult, L4, and L3 worms were counted to ensure that only the F_1_ generation (and the P_0_ worm) is counted and avoid confusion with the younger F_2_ generation. Thus, brood size counts were termed a ‘relative’ rather than absolute brood size. For statistics, Student's *t*-test was used to determine significance. For regression analysis, trendline function in Microsoft Excel was used, and a Pearson's correlation coefficient (R) was calculated for each regression. Student's *t*-test was performed to analyze significance of the regression.

### Lifespan assay

L3 worms were picked onto NGM plates or NGT-3D supplemented with 120 µM 5-fluoro-2′-deoxyuridine (TCI, Japan), and day 1 of the assay started 24 h later. A total of 50 worms on NGM plates and 44 worms on NGT-3D were counted. Viability was checked by tapping worms with a platinum wire pick for NGM plates, or observation of any worm movement over a 6 h time period for NGT-3D. Log-rank test analysis was used to determine significance.

## References

[BIO015743C1] Bargmann C. I. (2006). Chemosensation in C. elegans. In *WormBook* (ed. The C. elegans Research Community), pp. 1-29, 10.1895/wormbook.1.123.1. PMC4781564 18050433

[BIO015743C2] Barrière A. and Félix M.-A. (2005). High local genetic diversity and low outcrossing rate in Caenorhabditis elegans natural populations. *Curr. Biol.* 15, 1176-1184. 10.1016/j.cub.2005.06.022 16005289

[BIO015743C3] Beron C., Vidal-Gadea A. G., Cohn J., Parikh A., Hwang G. and Pierce-Shimomura J. T. (2015). The burrowing behavior of the nematode Caenorhabditis elegans: a new assay for the study of neuromuscular disorders. *Genes Brain Behav.* 14, 357-368. 10.1111/gbb.12217 25868909 PMC4444045

[BIO015743C4] Coburn C. M. and Bargmann C. I. (1996). A putative cyclic nucleotide-gated channel is required for sensory development and function in C. elegans. *Neuron* 17, 695-706. 10.1016/S0896-6273(00)80201-9 8893026

[BIO015743C5] Coburn C. M., Mori I., Ohshima Y. and Bargmann C. I. (1998). A cyclic nucleotide-gated channel inhibits sensory axon outgrowth in larval and adult Caenorhabditis elegans: a distinct pathway for maintenance of sensory axon structure. *Development* 125, 249-258. 9486798 10.1242/dev.125.2.249

[BIO015743C6] Collet J., Spike C. A., Lundquist E. A., Shaw J. E. and Herman R. K. (1998). Analysis of osm-6, a gene that affects sensory cilium structure and sensory neuron function in Caenorhabditis elegans. *Genetics* 148, 187-200. 9475731 10.1093/genetics/148.1.187 PMC1459801

[BIO015743C7] Epstein H. F., Waterston R. H. and Brenner S. (1974). A mutant affecting the heavy chain of myosin in Caenorhabditis elegans. *J. Mol. Biol.* 90, 291-300. 10.1016/0022-2836(74)90374-X 4453018

[BIO015743C8] Ferris H. and Hieb W. F. (2015). Ellsworth C. Dougherty: a pioneer in the selection of Caenorhabditis elegans as a model organism. *Genetics* 200, 991-1002. 10.1534/genetics.115.178913 26272995 PMC4574257

[BIO015743C9] Gray J. M., Hill J. J. and Bargmann C. I. (2005). A circuit for navigation in Caenorhabditis elegans. *Proc. Natl. Acad. Sci. USA* 102, 3184-3191. 10.1073/pnas.0409009101 15689400 PMC546636

[BIO015743C10] Kenyon C., Chang J., Gensch E., Rudner A. and Tabtiang R. (1993). A C. elegans mutant that lives twice as long as wild type. *Nature* 366, 461-464. 10.1038/366461a0 8247153

[BIO015743C11] Kim D. H., Feinbaum R., Alloing G., Emerson F. E., Garsin D. A., Inoue H., Tanaka-Hino M., Hisamoto N., Matsumoto K., Tan M.-W. et al. (2002). A conserved p38 MAP kinase pathway in Caenorhabditis elegans innate immunity. *Science* 297, 623-626. 10.1126/science.1073759 12142542

[BIO015743C12] Kimura K. D., Tissenbaum H. A., Liu Y. and Ruvkun G. (1997). daf-2, an insulin receptor-like gene that regulates longevity and diapause in Caenorhabditis elegans. *Science* 277, 942-946. 10.1126/science.277.5328.942 9252323

[BIO015743C13] Kwon N., Pyo J., Lee S.-J. and Je J. H. (2013). 3-D worm tracker for freely moving C. elegans. *PLoS ONE* 8, e57484 10.1371/journal.pone.0057484 23437394 PMC3578814

[BIO015743C14] Lewis J. A. and Fleming J. T. (1995). Basic culture methods. In *Caenorhabditis elegans: Modern Biological Analysis of an Organism*, Vol. 48 (ed. Epstein H. F. S. and Shakes D. C.), pp. 4-27. San Diego, CA: Academic Press.

[BIO015743C15] Ou G., Koga M., Blacque O. E., Murayama T., Ohshima Y., Schafer J. C., Li C., Yoder B. K., Leroux M. R. and Scholey J. M. (2007). Sensory ciliogenesis in Caenorhabditis elegans: assignment of IFT components into distinct modules based on transport and phenotypic profiles. *Mol. Biol. Cell* 18, 1554-1569. 10.1091/mbc.E06-09-0805 17314406 PMC1855012

[BIO015743C16] Raizen D. M., Lee R. Y. and Avery L. (1995). Interacting genes required for pharyngeal excitation by motor neuron MC in Caenorhabditis elegans. *Genetics* 141, 1365-1382. 8601480 10.1093/genetics/141.4.1365 PMC1206873

[BIO015743C17] Richmond J. E., Davis W. S. and Jorgensen E. M. (1999). UNC-13 is required for synaptic vesicle fusion in C. elegans. *Nat. Neurosci.* 2, 959-964. 10.1038/14755 10526333 PMC2585767

[BIO015743C18] Tanaka-Hino M., Sagasti A., Hisamoto N., Kawasaki M., Nakano S., Ninomiya-Tsuji J., Bargmann C. I. and Matsumoto K. (2002). SEK-1 MAPKK mediates Ca2+ signaling to determine neuronal asymmetric development in Caenorhabditis elegans. *EMBO Rep.* 3, 56-62. 10.1093/embo-reports/kvf001 11751572 PMC1083920

[BIO015743C19] Van Voorhies W. A., Fuchs J. and Thomas S. (2005). The longevity of Caenorhabditis elegans in soil. *Biol. Lett.* 1, 247-249. 10.1098/rsbl.2004.0278 17148178 PMC1626236

